# Supplementary material for: Activity of the mammalian DNA transposon piggyBat from Myotis lucifugus is restricted by its own transposon ends
Source: Nat Commun. 2025 Jan 7;16:458. doi: 10.1038/s41467-024-55784-9 (PMC11707139; doi:10.1038/s41467-024-55784-9)

Activity of the mammalian DNA transposon *piggyBat* from *Myotis lucifugus* is restricted by its own transposon ends

Separated traces for Fig. 1d:

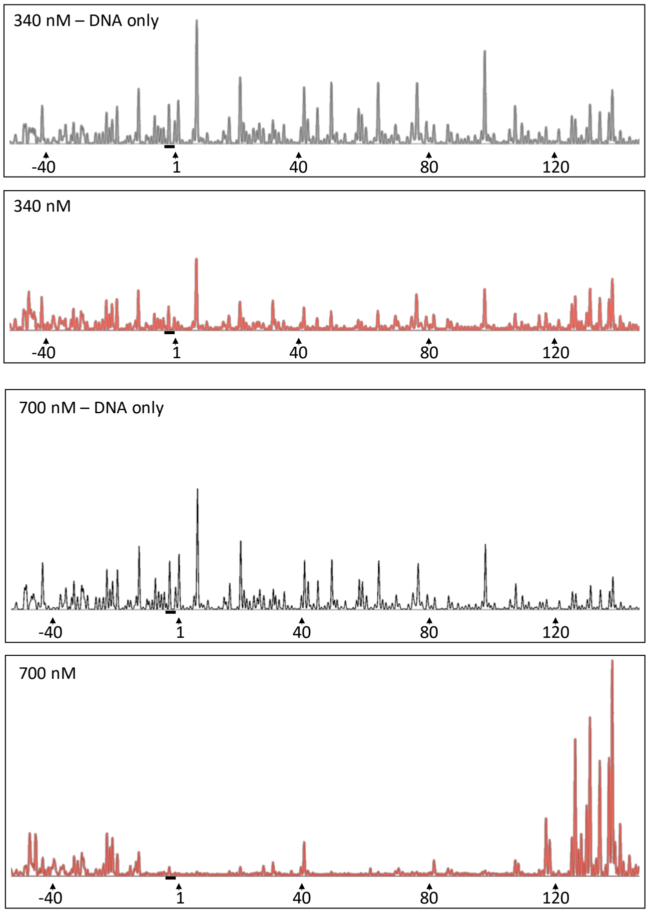

Separated traces for Fig. 1e:

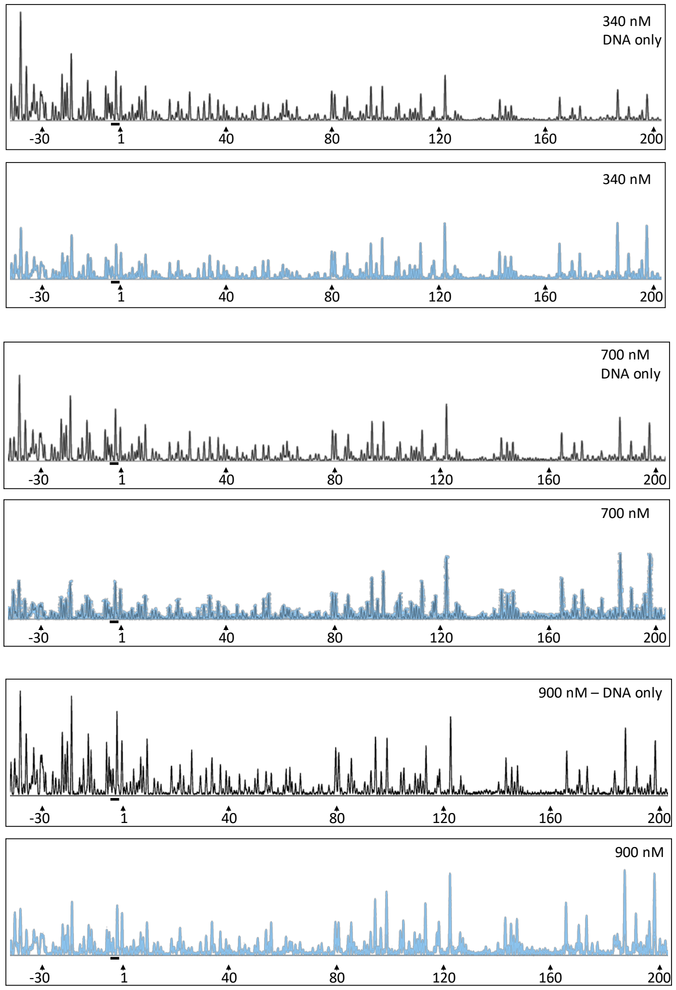

Separated traces for Fig. 1f:

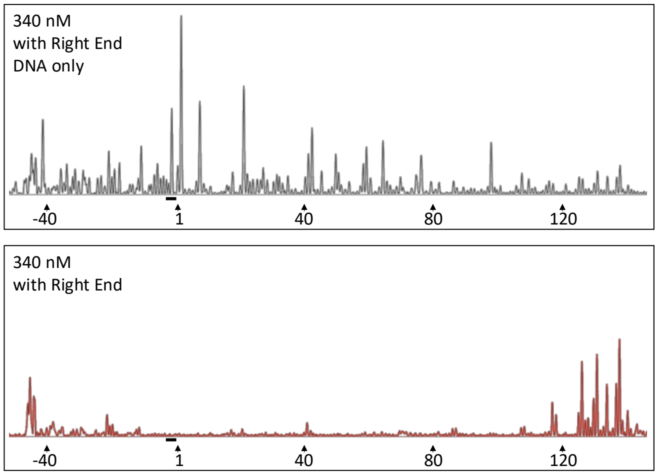

Separated traces for Fig. 1g:

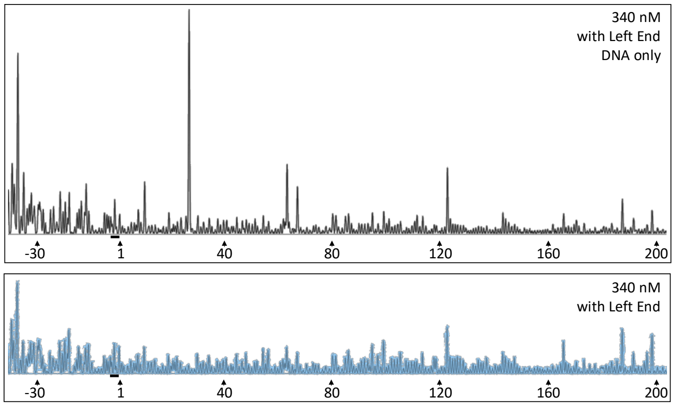

Supplementary Figure 1. Separated electropherograms for footprinting experiments to assess pBat binding to LE153 and RE208. Black traces are DNA alone. Red traces are those with labelled LE and blue are those with labelled RE.

Example 1: Starting with complex formed with RE100, titrating in LE88:

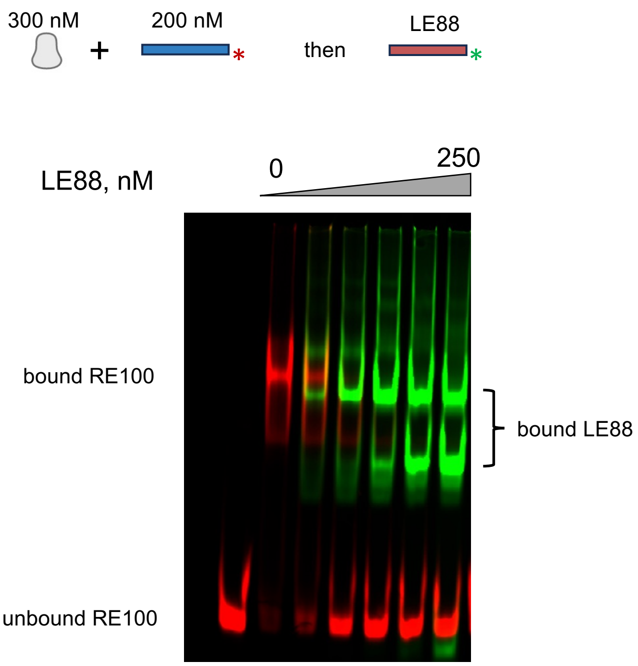

Example 2: Starting with complex formed with LE88, titrating RE100:

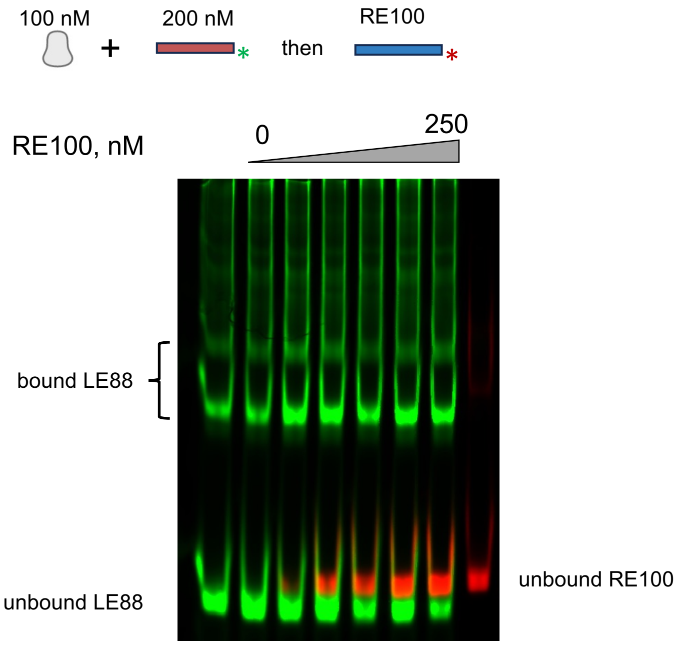

Supplementary Figure 2. EMSA experiments to detect synaptic complexes. A range of experimental conditions were tested (n>10, only two are shown here), but no evidence was found for simultaneous LE88+RE100 binding.

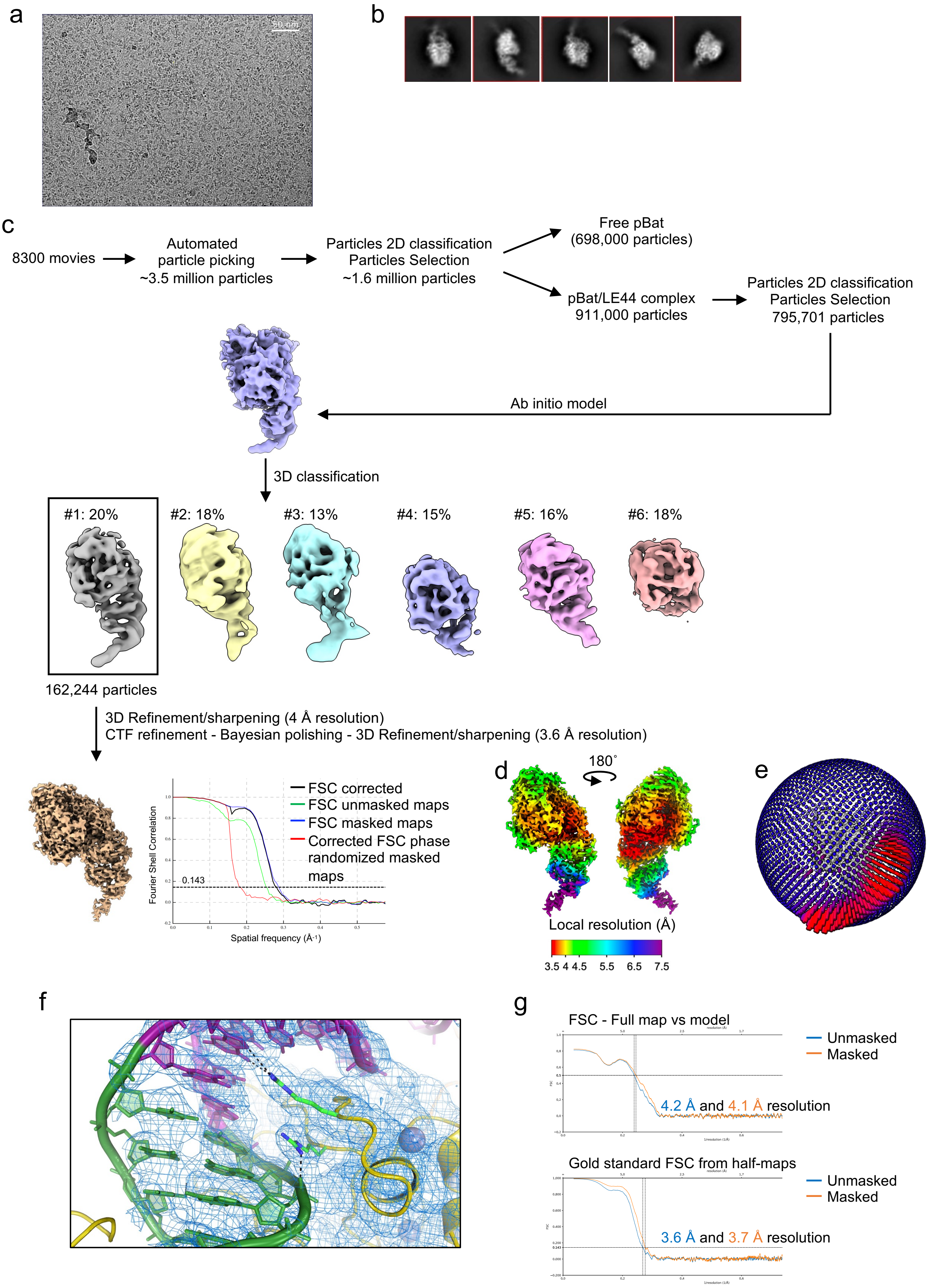

Supplementary Figure 3. Cryo-EM data processing workflow. (a) Representative cryo-EM micrograph of the pBat/LE44 sample. (b) Sample 2D classes. (c) Computational workflow used for structure determination, including final sharpened reconstruction and Fourier shell correlation (SC) curves. (d) Resolution distribution of the final reconstruction from RELION. (e) Distribution of particle views used for the final reconstruction. (f) Sample density. (g) FSC curves.

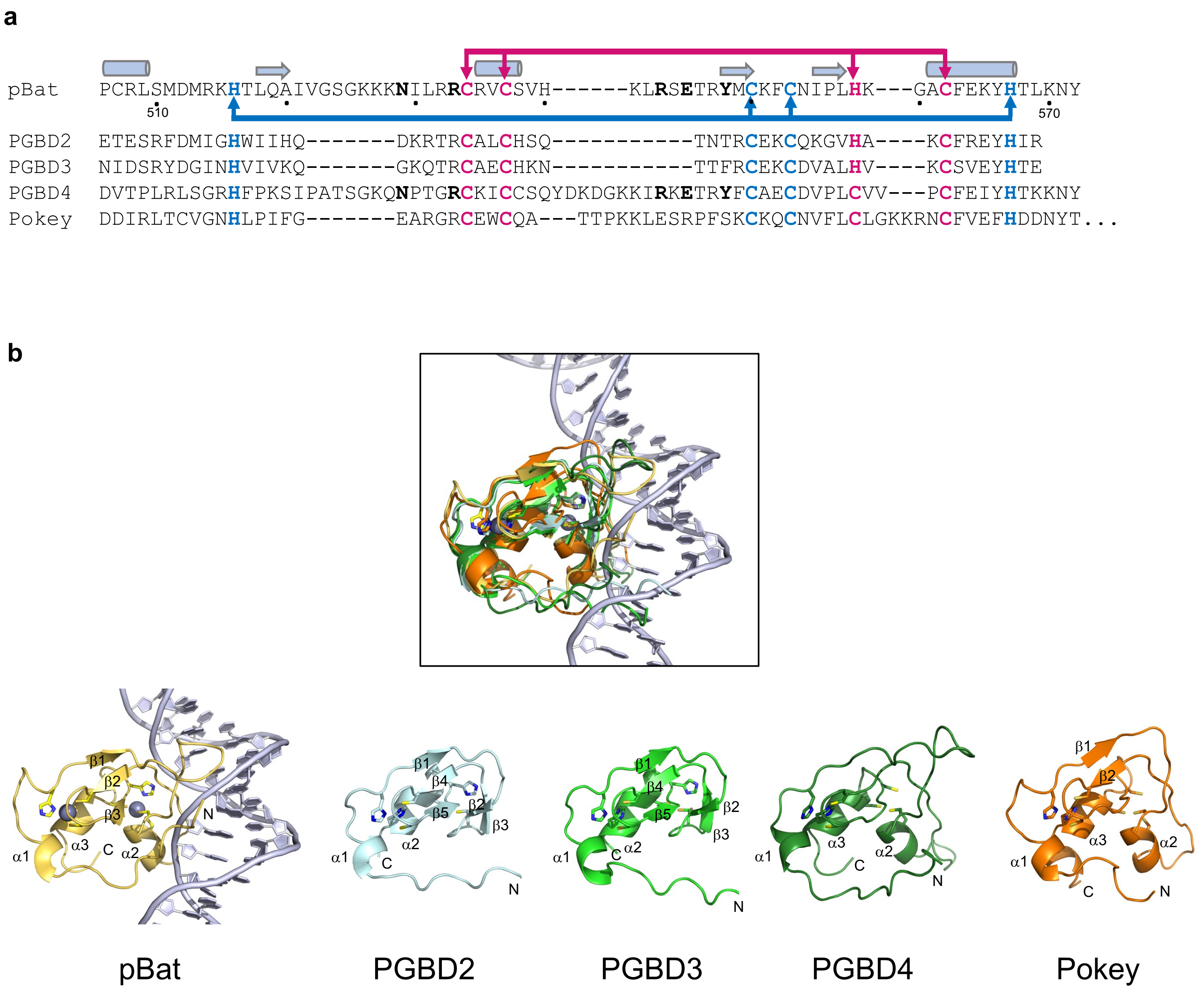

Supplementary Figure 4. Analysis of pBat cysteine-rich domain (CRD). (a) Amino acid sequence alignment of the CRDs of pBat with human PGBD2, PGBD3, PGBD4, and the Pokey transposase. The secondary structure of pBat is shown with cylinders for α-helices and arrows for β-strands. The ligands for the two Zn2+ ions are shown in blue and in pink. Highlighted in bold for pBat are residues that make direct contact with DNA and that are conserved in PGBD4. (b) Structural alignment of the experimentally determined pBat/DNA structure with the AlphaFold2 predictions for human PGBD2, PGBD3, PGBD4, and the Pokey transposase.

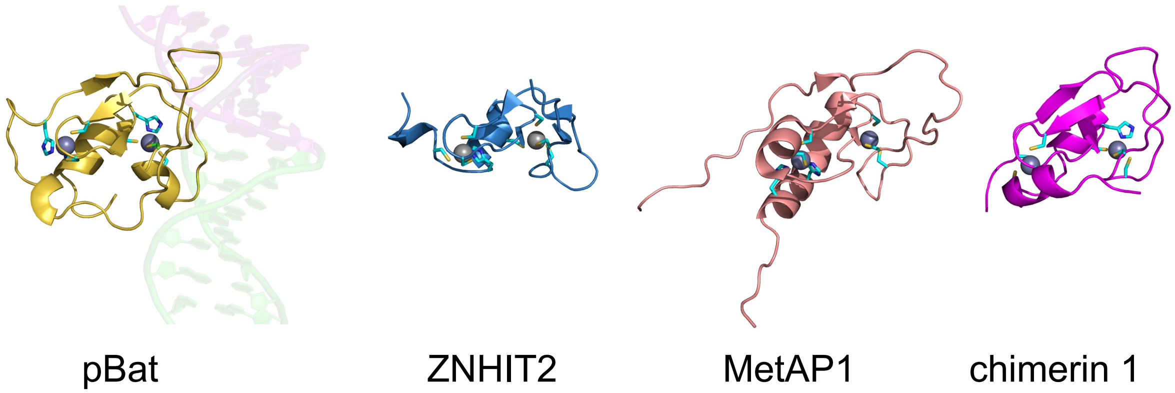

Supplementary Figure 5. Structurally homologous domains to the pBat CRD identified by DALI. ZNHIT2: PDB ID 1X4S. MetAP1: PDB ID 7SEK. chimerin 1: PDB ID 3CXL. Alignments were performed in Pymol.

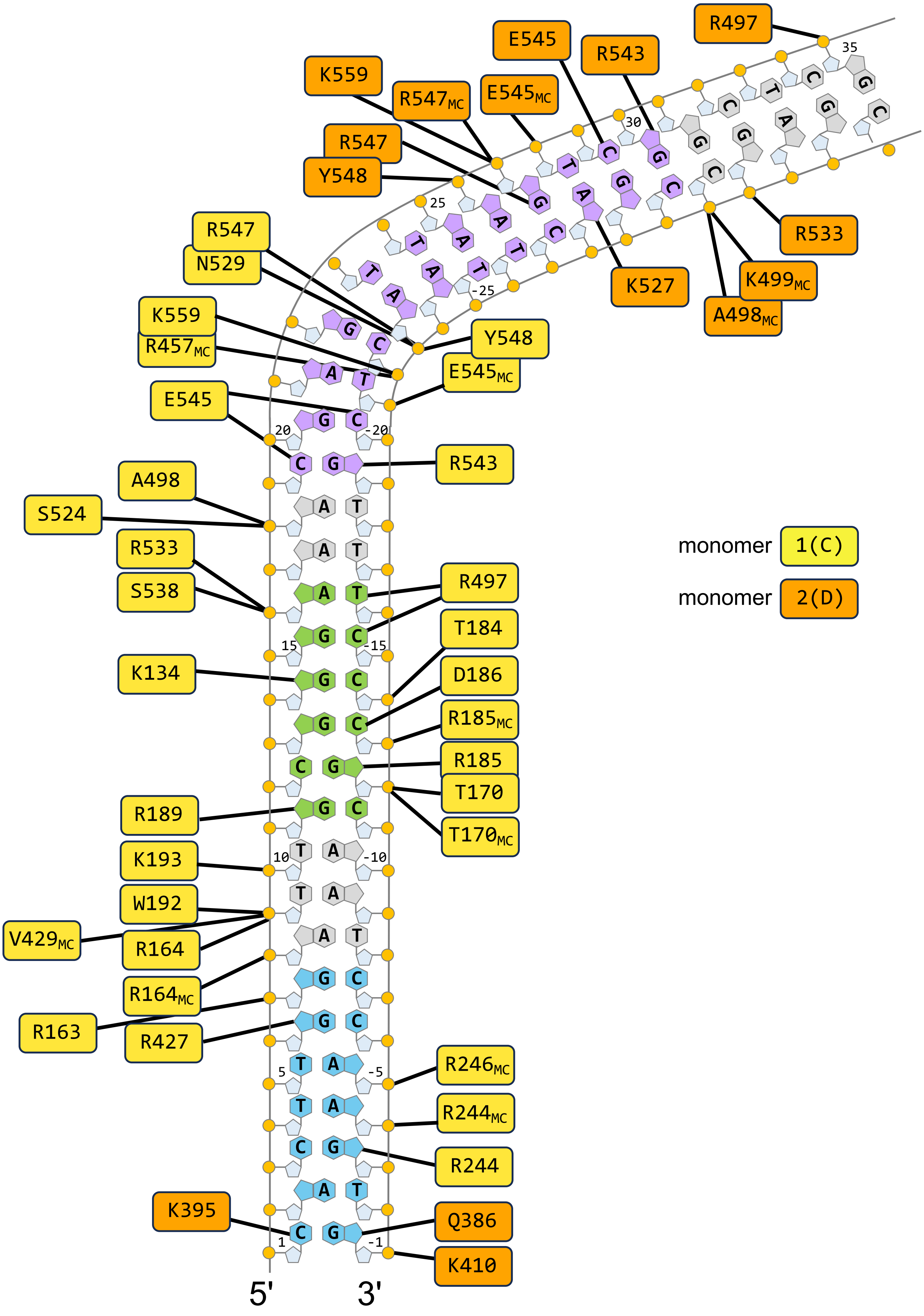

Supplementary Figure 6. Schematic of observed interactions between pBat and LE44. Phosphates are shown as orange circles; basepair and domain coloring ("C" and "D" refer to the chain IDs in PDB ID 9C0F) is as in Figure 4a. MC= main chain interaction

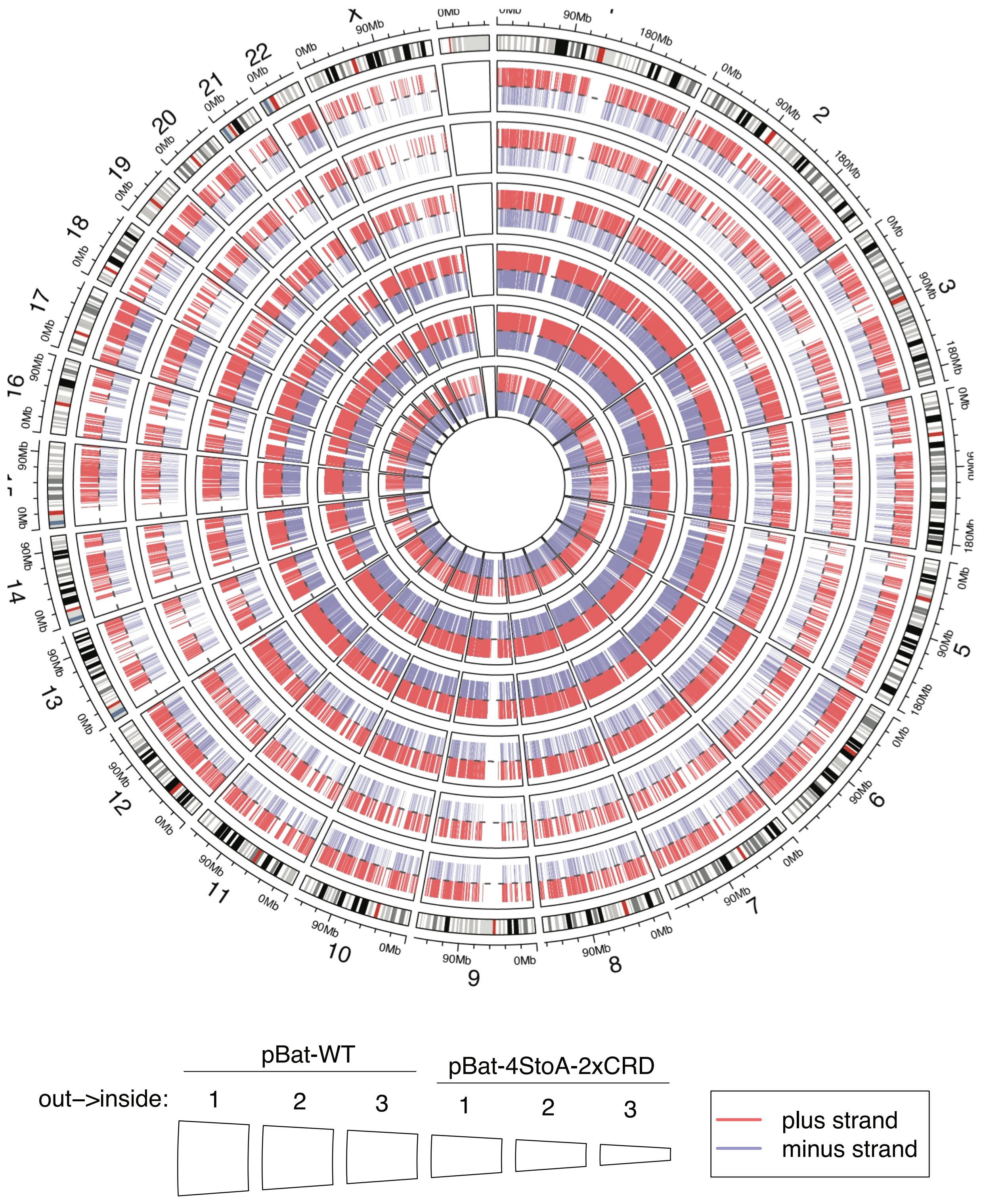

Supplementary Figure 7. pBat insertion sites throughout the genome. The genome-wide distributions of insertion peaks for all replicates are shown for pBat-WT and pBat-4StoA-2xCRDV1. Circplots were calculated according to Krzywinski et al[73]. From inside to outside: pBat-WT (replicates 1-3), pBat-4StoA-2xCRDV1 (replicates 1-3) self-reporting transcripts driven from the genomic insertions are shown on the + (red) and – (blue) strand. Insertions are detected on all chromosomes except for the Y chromosome, which has been lost in HCT116 cells.

Supplementary Table 1

Cryo-EM data collection, refinement and validation statistics

	pBat bound to LE44 (EMDB-45082) (PDB 9C0F)
Data collection and processing	
Magnification	105 kx
Voltage (kV)	300
Electron exposure (e–/Å^2)	48.8
Defocus range (μm)	-0.8 to -2.0
Pixel size (Å)	0.43
Symmetry imposed	none
Initial particle images (no.)	795,701
Final particle images (no.)	162,244
Map resolution (Å)	3.6
FSC threshold	0.143
Map resolution range (Å)	3.1-4.2
Refinement	
Initial model used (PDB code)	none
Model resolution (Å)	3.3
FSC threshold	0.143
Model resolution range (Å)	3.1-4.2
Map sharpening B factor (Å^2)	-210.6
Model composition	
Non-hydrogen atoms	9507
Protein residues	970
Ligands	dsDNA (70 nucleotides)<br>4 x Zn^{2+}
B factors (Å^2)	
Protein	70.90
Ligand (DNA)	95.97
Ligand (Zn)	94.17
R.m.s. deviations	
Bond lengths (Å)	0.028
Bond angles (°)	1.87
Validation	
MolProbity score	1.16
Clashscore	2.12
Poor rotamers (%)	0.11
Ramachandran plot	
Favored (%)	96.97
Allowed (%)	2.41
Disallowed (%)	0.63

Uncropped scans for gels in Supplementary Figure 2

Example 1 (N.B. images are reversed in figure and dashed box shows lanes displayed):

Cy5 (LE88)

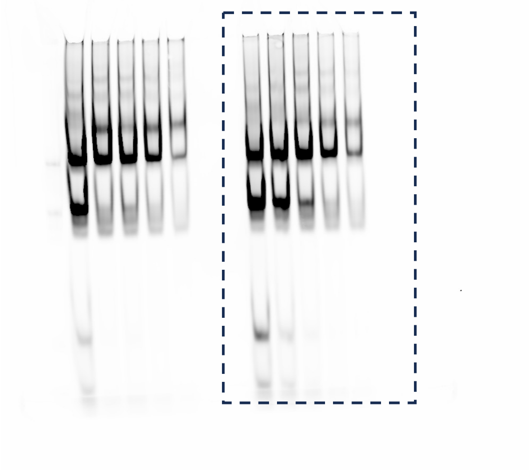

Cy3 (RE100)

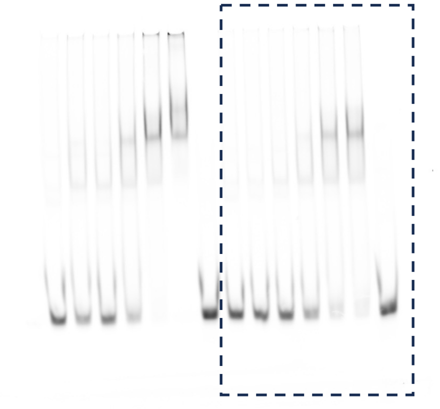

Example 2 (dashed box shows lanes displayed):

Cy3 (RE100)

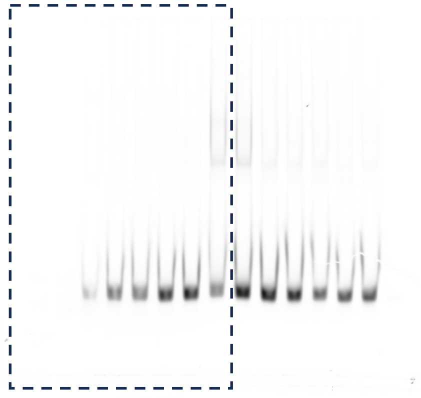

Cy5 (LE88)

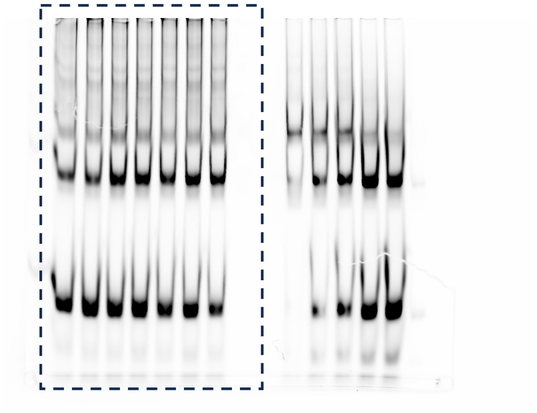

Supplement: Supplementary file 1 — Supplementary Information [file 41467_2024_55784_MOESM1_ESM.pdf]
