# Supplementary material for: Activity of the mammalian DNA transposon piggyBat from Myotis lucifugus is restricted by its own transposon ends
Source: Nat Commun. 2025 Jan 7;16:458. doi: 10.1038/s41467-024-55784-9 (PMC11707139; doi:10.1038/s41467-024-55784-9)
Supplement: Supplementary file 2 — Description of Additional Supplementary Files [file 41467_2024_55784_MOESM2_ESM.pdf]

Description of Additional Supplementary Files

Supplementary Data 1: Sequences of oligonucleotides used
