# Supplementary material for: Activity of the mammalian DNA transposon piggyBat from Myotis lucifugus is restricted by its own transposon ends
Source: Nat Commun. 2025 Jan 7;16:458. doi: 10.1038/s41467-024-55784-9 (PMC11707139; doi:10.1038/s41467-024-55784-9)
Supplement: Supplementary file 4 — Reporting Summary [file 41467_2024_55784_MOESM4_ESM.pdf]

nature portfolio

Corresponding author(s): Fred Dyda

Last updated by author(s): Nov 30, 2024

Reporting Summary

Nature Portfolio wishes to improve the reproducibility of the work that we publish. This form provides structure for consistency and transparency in reporting. For further information on Nature Portfolio policies, see our Editorial Policies and the Editorial Policy Checklist.

Statistics

For all statistical analyses, confirm that the following items are present in the figure legend, table legend, main text, or Methods section.

n/a	Confirmed	
☐	☒	The exact sample size (*n*) for each experimental group/condition, given as a discrete number and unit of measurement
☐	☒	A statement on whether measurements were taken from distinct samples or whether the same sample was measured repeatedly
☐	☒	The statistical test(s) used AND whether they are one- or two-sided<br>*Only common tests should be described solely by name; describe more complex techniques in the Methods section.*
☒	☐	A description of all covariates tested
☒	☐	A description of any assumptions or corrections, such as tests of normality and adjustment for multiple comparisons
☐	☒	A full description of the statistical parameters including central tendency (e.g. means) or other basic estimates (e.g. regression coefficient) AND variation (e.g. standard deviation) or associated estimates of uncertainty (e.g. confidence intervals)
☐	☒	For null hypothesis testing, the test statistic (e.g. *F*, *t*, *r*) with confidence intervals, effect sizes, degrees of freedom and *P* value noted<br>*Give P values as exact values whenever suitable.*
☒	☐	For Bayesian analysis, information on the choice of priors and Markov chain Monte Carlo settings
☒	☐	For hierarchical and complex designs, identification of the appropriate level for tests and full reporting of outcomes
☒	☐	Estimates of effect sizes (e.g. Cohen's *d*, Pearson's *r*), indicating how they were calculated

Our web collection on statistics for biologists contains articles on many of the points above.

Software and code

Policy information about availability of computer code

Data collection	SerialEM (3.8) was used to collect cryo-EM data
Data analysis	RELION (4.0.1), CTFFind 4.1.14, Topaz 0.2.5, DeepEMhancer, COOT 0.9 EL; Phenix 1.19.2; UCSF Chimera (1.13); UCSF ChimeraX (1.3), PyMol (2.5.2); Rosetta (3.12)<br>Prism (10.2.2);<br>cutadapt (v4.4),bowtie2 (v2.5.1),bedtools suite (v2.31.0),weblogo (v3.7.12), Integrative Genomics Viewer (v2.16.2)

For manuscripts utilizing custom algorithms or software that are central to the research but not yet described in published literature, software must be made available to editors and reviewers. We strongly encourage code deposition in a community repository (e.g. GitHub). See the Nature Portfolio guidelines for submitting code & software for further information.

Data

Policy information about availability of data

All manuscripts must include a data availability statement. This statement should provide the following information, where applicable:

- Accession codes, unique identifiers, or web links for publicly available datasets
- A description of any restrictions on data availability
- For clinical datasets or third party data, please ensure that the statement adheres to our policy

The atomic coordinates of the pBat/LE44 complex have been deposited in the protein data bank (PDB) with the accession code 9C0F [https://doi.org/10.2210/

pdb9c0f/pdb]. The EM map has been deposited in the Electron Microscopy Data Bank (EMDB) with accession code EMD-45082 [https://www.ebi.ac.uk/emdb/EMD-45082]. Next-generation sequencing data are available at the Gene Expression Omnibus (GEO) GSE245531 [https://www.ncbi.nlm.nih.gov/geo/query/acc.cgi?acc=GSE245531].

Research involving human participants, their data, or biological material

Policy information about studies with human participants or human data. See also policy information about sex, gender (identity/presentation), and sexual orientation and race, ethnicity and racism.

Reporting on sex and gender	This work did not involve human participants, their data, or biological material.
Reporting on race, ethnicity, or other socially relevant groupings	This work did not involve human participants, their data, or biological material.
Population characteristics	This work did not involve human participants, their data, or biological material.
Recruitment	This work did not involve human participants, their data, or biological material.
Ethics oversight	This work did not involve human participants, their data, or biological material.

Note that full information on the approval of the study protocol must also be provided in the manuscript.

Field-specific reporting

Please select the one below that is the best fit for your research. If you are not sure, read the appropriate sections before making your selection.

☒ Life sciences ☐ Behavioural & social sciences ☐ Ecological, evolutionary & environmental sciences

For a reference copy of the document with all sections, see nature.com/documents/nr-reporting-summary-flat.pdf

Life sciences study design

All studies must disclose on these points even when the disclosure is negative.

Sample size	Most experiments entailed four biological replicates, although some were performed in biological triplicate or duplicate as noted in the figure legends. No statistical methods were used to predetermine sample size; rather, sample size was selected based on standards and precedents in the literature, generally seen as sufficient to approximate the mean per experimental measurement. Sufficient cryo-EM data were collected to achieve adequate map resolution for model building
Data exclusions	No data were excluded from the analyses.
Replication	All biochemical assays and cell-based assays were repeated at least twice, and most experimental conditions were evaluated multiple times in various combinations. All data could be reproduced.
Randomization	Randomization was not relevant to this study because our study did not involve the allocation of samples/organisms/participants into groups.
Blinding	Blinding was not relevant to this study. Investigators were not blinded to group allocation because group allocation was not involved in our study.

Reporting for specific materials, systems and methods

We require information from authors about some types of materials, experimental systems and methods used in many studies. Here, indicate whether each material, system or method listed is relevant to your study. If you are not sure if a list item applies to your research, read the appropriate section before selecting a response.

Materials & experimental systems

n/a	Involved in the study	
☒	☐	Antibodies
☐	☒	Eukaryotic cell lines
☒	☐	Palaeontology and archaeology
☒	☐	Animals and other organisms
☒	☐	Clinical data
☒	☐	Dual use research of concern
☒	☐	Plants

Methods

n/a	Involved in the study	
☒	☐	ChIP-seq
☒	☐	Flow cytometry
☒	☐	MRI-based neuroimaging

Eukaryotic cell lines

Policy information about cell lines and Sex and Gender in Research

Cell line source(s)	HEK293-T/17 cells were obtained from ATCC (#CRL-11268). HCT116 cells were obtained from ATCC, CCL-247
Authentication	Cells were authenticated by the provider ATCC.
Mycoplasma contamination	Cell lines were not tested for mycoplasma contamination.
Commonly misidentified lines (See ICLAC register)	No commonly misidentified cell lines were used in this study.

Plants

Seed stocks	*Report on the source of all seed stocks or other plant material used. If applicable, state the seed stock centre and catalogue number. If plant specimens were collected from the field, describe the collection location, date and sampling procedures.*
Novel plant genotypes	*Describe the methods by which all novel plant genotypes were produced. This includes those generated by transgenic approaches, gene editing, chemical/radiation-based mutagenesis and hybridization. For transgenic lines, describe the transformation method, the number of independent lines analyzed and the generation upon which experiments were performed. For gene-edited lines, describe the editor used, the endogenous sequence targeted for editing, the targeting guide RNA sequence (if applicable) and how the editor was applied.*
Authentication	*Describe any authentication procedures for each seed stock used or novel genotype generated. Describe any experiments used to assess the effect of a mutation and, where applicable, how potential secondary effects (e.g. second site T-DNA insertions, mosiacism, off-target gene editing) were examined.*
